# Ventricular tachycardia arising from His‐bundle

**DOI:** 10.1002/joa3.12855

**Published:** 2023-04-13

**Authors:** Sou Takenaka, Akihiko Ueno, Ayano Enzan, Masayoshi Sakakibara

**Affiliations:** ^1^ Department of Cardiology IMS Katsushika Heart Center Tokyo Japan; ^2^ Division of Cardiology Kawasaki Medical School Kurashiki Japan

**Keywords:** ablation, His‐bundle, His‐Purkinje system, ventricular tachycardia

## Abstract

During tachycardia, His‐bundle potentials preceded Purkinje potentials. When the radiofrequency application was performed at a site where Purkinje potentials could be recorded slightly more peripherally than His‐bundle potentials, tachycardia temporarily stopped, but was quickly followed by tachycardia with left‐axis deviated because of the complication of the left anterior fascicular block.

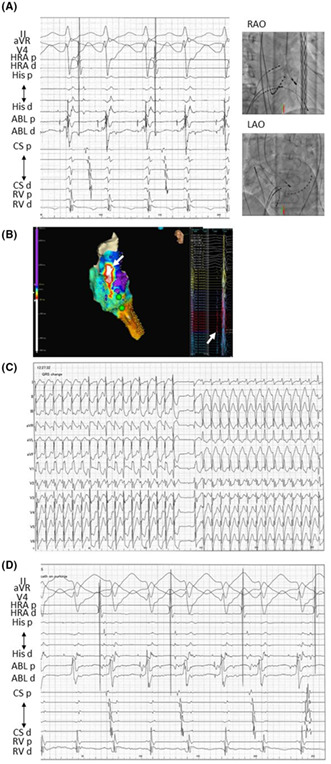

A 65‐year‐old man visited our hospital complaining of palpitations. The ECG revealed right bundle branch block (RBBB) type wide QRS tachycardia (Figure 1A), which was not stopped by 20 mg‐ATP infusion and had to be defibrillated electrically. The QRS complex in sinus rhythm was very similar to the one observed during this tachycardia (Figure 1A). He continued to experience tachycardia attacks, which were stopped by a 60 mg‐ATP infusion suddenly without prolongation of R‐R interval.

**FIGURE 1:**
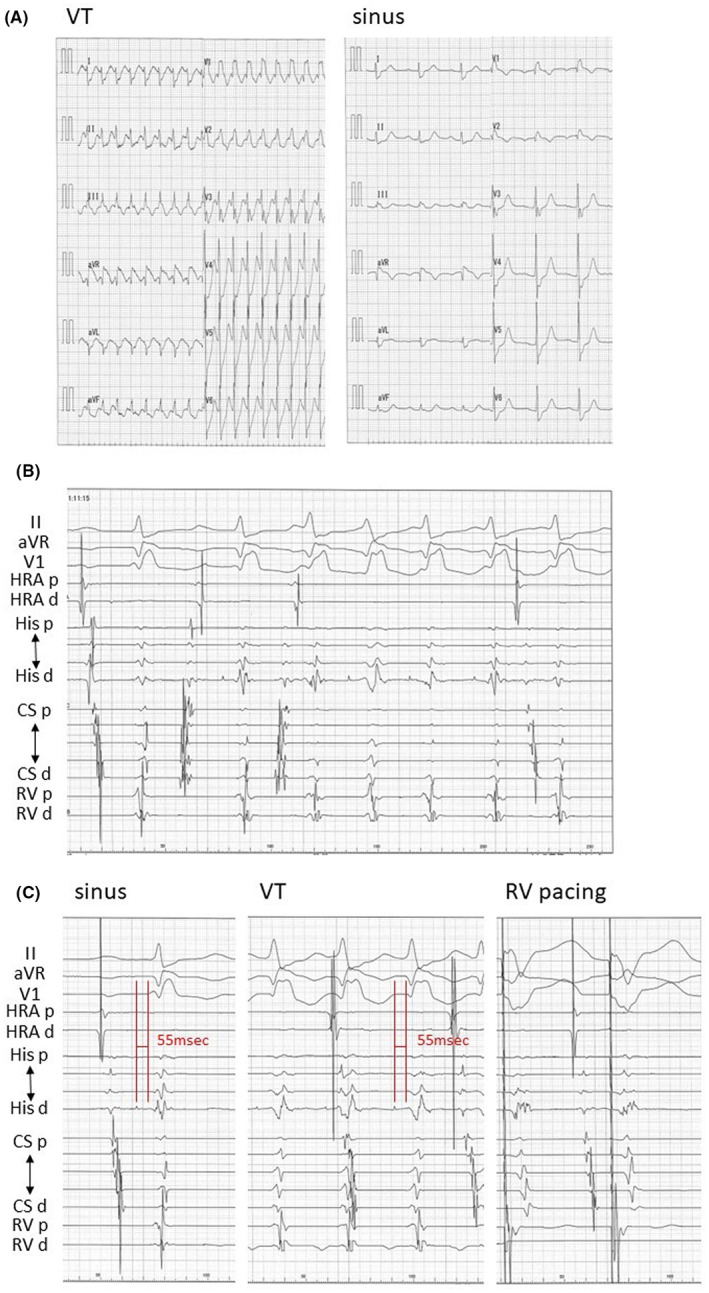
(A) ECG during tachycardia and sinus rhythm at the first session. The QRS complex was an approximation. (B) An intra‐cardiac electrocardiogram was obtained when tachycardia occurred. His‐bundle potentials were the first. (C) Intra‐cardiac electrocardiogram during sinus rhythm and tachycardia. During sinus rhythm, the time from His‐bundle potential to QRS complex was similar. CS, coronary sinus; d, distal; p, proximal; His, His‐bundle; HRA, high right atrium; RV, right ventricle; VT, ventricular tachycardia.

Tachycardia occurred spontaneously, but not with pacing maneuvers, during the first session. Prior to the QRS complex, His‐bundle potentials were recorded and there was no ventriculo‐atrial conduction. (Figure 1B). The time from His‐bundle potential to QRS complex during sinus rhythm was similar to that during tachycardia (55 ms) (Figure 1C). In addition, there should be no retrograde conduction between the ventricle and His‐bundle during right ventricular pacing (Figure 1C). These electrophysiological findings demonstrated that this tachycardia was not bundled with branch reentry tachycardia. During tachycardia, His‐bundle potentials preceded Purkinje potentials (Figure 2A). The activation mapping during VT revealed the earliest site was above the His‐bundle region when the timing of His and Purkinje potentials were annotated as local activation time (Figure 2B). When the radiofrequency application was performed at a site where Purkinje potentials could be recorded slightly more peripherally than His‐bundle potentials (Figure 2A), tachycardia temporarily stopped, but was quickly followed by tachycardia with left‐axis deviated (LAD) (Figure 2C) because of the complication of the left anterior fascicular block. During this tachycardia with RBBB‐type and LAD QRS complex, His‐bundle potential also preceded Purkinje potential and the QRS complex (Figure 2D). We also had atrio‐ventricular node slow pathway modification, but this did not result in a complete cure for the tachycardia. The radiofrequency application was not delivered at the aortic cusp because the potential of the aortic cusp was not so early. We abandoned the first session because pilsicainide was effective for this tachycardia, and medical therapy was begun.

**FIGURE 2:**
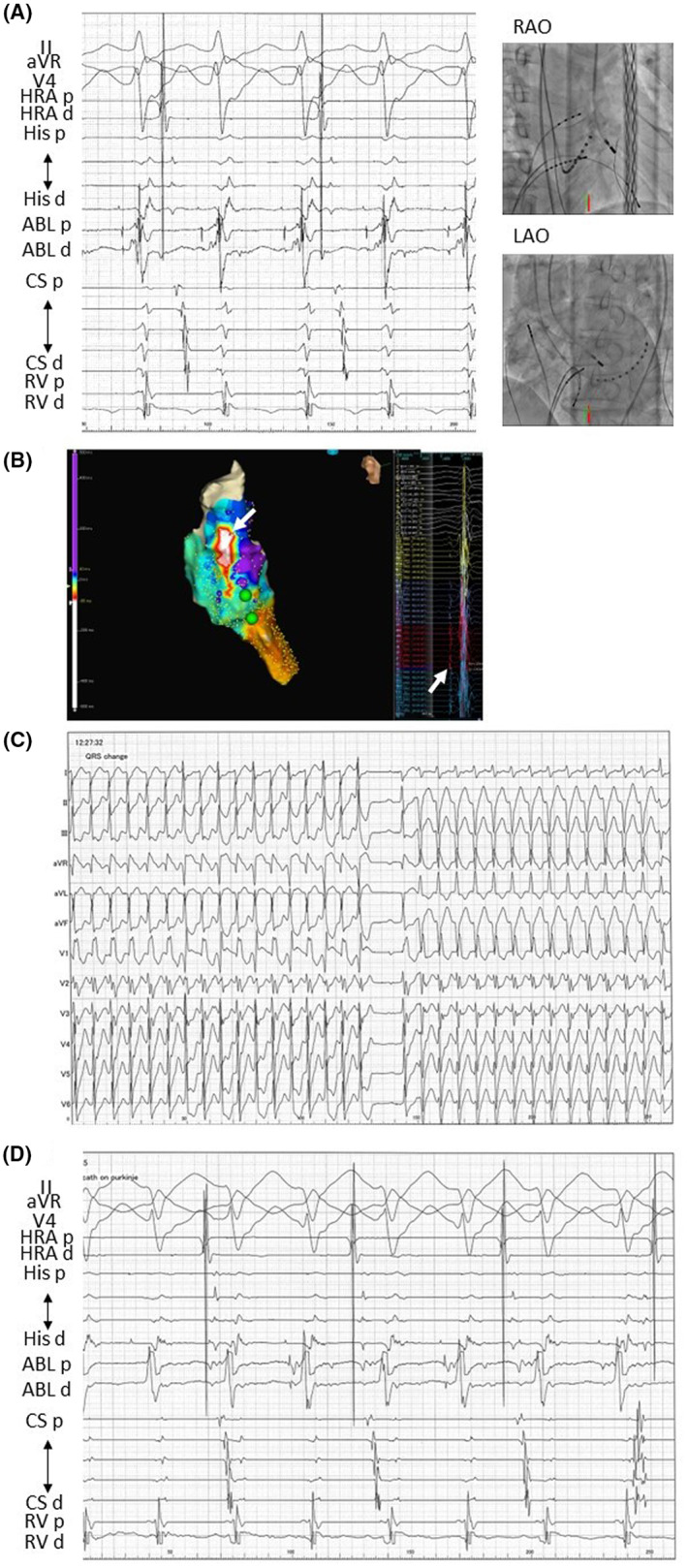
(A) Intra‐cardiac ECG during tachycardia. Purkinje potentials were recorded on an ablation catheter. The preceding HH interval defined the VV interval during tachycardia. When ablation was performed on the conduction system distal to His, the order of excitation of His potentials was proximal to distal. (B) The activation mapping during VT. The earliest site (arrow) was above the His‐bundle region when the timing of His and Purkinje potentials were annotated as local activation time. Green tags were where the application was attempted. (C) ECG during application. Tachycardia temporarily stopped, but was quickly followed by tachycardia with LAD. (D) During the tachycardia with QRS complex of RBBB‐type and LAD, His‐bundle potential also preceded Purkinje potential and QRS complex. ABL, ablation catheter; CS, coronary sinus; d, distal; His, His‐bundle; HRA, high right atrium; LAO, left anterior oblique; p, proximal; RAO, right anterior oblique; RV, right ventricle.

He returned to our emergency room 2 months later with tachycardia. This tachycardia was repetitive, and the ECG revealed that the QRS complex was wide and had a left bundle branch block type, which was different from the QRS complex in sinus rhythm (Figure 3A). The electrophysiologic findings during the second session revealed the followings: (1) since no His‐bundle potentials were recorded during RV pacing, there was no conduction transmitted from the left bundle branch to His‐bundle; (2) he also had RBBB, and there is no conduction traveling down the right bundle branch. Therefore, this tachycardia was diagnosed as ventricular tachycardia (VT), which has a completely different mechanism than RBBB‐type tachycardia. The anterior wall of the right ventricle was discovered to be the earliest site by three‐dimensional mapping (Figure 3B). After radiofrequency application at the earliest site, this tachycardia was terminated (Figure 3B). However, presently, a wide QRS tachycardia with RBBB‐type and LAD spontaneously occurred (Figure 3C), which was stopped by antitachycardia pacing. He was started on amiodarone and had an implantable cardioverter defibrillator (ICD) implantation.

**FIGURE 3:**
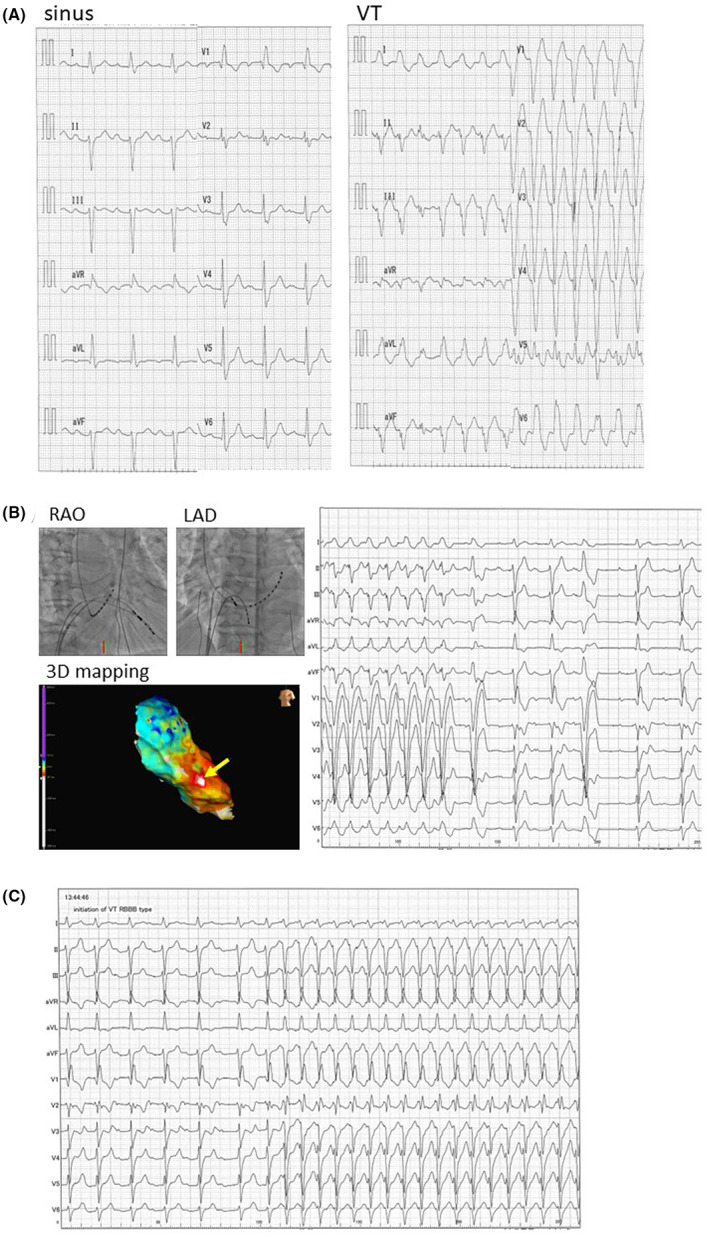
(A) ECG during tachycardia and sinus rhythm at the second session. During tachycardia, the QRS complex was broad and had a left bundle branch block. (B) Mapping within the RV. Tachycardia was stopped after ablation at the earliest site on the anterior wall of the RV. (C) A wide QRS tachycardia with RBBB‐type and LAD occurred spontaneously immediately following the session. LAO, left anterior oblique; RAO, right anterior oblique.

One month after the second session, he was consulted because of an ICD shock. Therefore, we performed His‐bundle ablation. He had an atrio‐ventricular block and all ventricular pacing. Now, he had no tachycardia, and could discontinue his antiarrhythmic medications.

During RBBB‐type tachycardia, the His‐bundle was activated before the QRS complex. In this case, conduction to the His‐Purkinje system was far greater than conduction to the ventricular myocardium, resulting in early His‐Purkinje activation, and a QRS complex similar to that seen in sinus rhythm.^1^, ^2^ The atrio‐ventricular relationship is the most useful electrocardiographic feature in distinguishing VT from supraventricular tachycardia. We discovered atrio‐ventricular dissociation during RBBB‐type tachycardia, so we labeled it as VT.

Junctional ectopic tachycardia is a narrow QRS tachycardia characterized by an irregular cycle length, sinus capture beats, periods of variable atrio‐ventricular and/or ventriculoatrial relationship, and ventriculoatrial dissociation.^3^ There were no sinus capture beats, and no variable atrio‐ventricular and/or ventriculoatrial relationship in the RBBB‐type wide QRS tachycardia. Junctional ectopic tachycardia might be ruled out.

In atrio‐ventricular node reentrant tachycardia (AVNRT) with upper common pathway, if accompanied by block from AVN to atria, the intra‐cardiac electrocardiography might show that His‐bundle potential preceded QRS complex and that QRS complex was the same during sinus rhythm, as in the present case. However, AVNRT might be ruled out because AVN slow pathway modification did not cure RBBB‐type wide QRS tachycardia.

No recordings of His bundle potentials during RV pacing could excluded an orthodromic reciprocating tachycardia with a concealed nodoventricular pathway with block from atrio‐ventricular node to atria. In a concealed nodefascisular pathway with block from AVN to atria, it could not be completely ruled out as with AVNRT. However, there had been no previous reports of an orthodromic reciprocating tachycardia with a concealed nodoventricular or nodofascicular pathway without retrograde conduction block.

This tachycardia was not stopped by 20 mg‐ATP infusion, and was stopped by 60 mg‐ATP. The antiadrenergic effects of ATP on the depolarization rate are abolished when the resting membrane potential is reduced. Therefore, ATP's antiadrenergic effects on the His‐Purkinje system are not absolute, but are dependent on the relative level of resting membrane potential.^4^ His‐Purkinje system has a shallower resting membrane potential, thus weakens the antiadrenergic effect.

In addition to those mentioned above, the reasons for considering RBBB‐type wide QRS tachycardia to be VT arising from His‐bundle are as follows: (1) His‐bundle potentials were significantly earlier during tachycardia than QRS complex, (2) The morphology of QRS complex and HV time in sinus rhythm and tachycardia were almost identical, which findings were also seen after he was complicated by left anterior fascicular block, (3) Ablation was performed on His‐bundle and no VT occurred.^5^

## CONFLICT OF INTEREST STATEMENT

Authors declare no conflict of interests for this article.
